# The pooled estimate of the total fertility rate in sub-Saharan Africa using recent (2010–2018) Demographic and Health Survey data

**DOI:** 10.3389/fpubh.2022.1053302

**Published:** 2023-01-26

**Authors:** Desalegn Tesfa, Sofonyas Abebaw Tiruneh, Alemayehu Digssie Gebremariam, Melkalem Mamuye Azanaw, Melaku Tadege Engidaw, Belayneh Kefale, Bedilu Abebe, Tsion Dessalegn, Mulu Tiruneh

**Affiliations:** ^1^Department of Public Health, College of Health Sciences, Debre Tabor University, Debre Tabor, Ethiopia; ^2^Department of Pharmacy, College of Health Sciences, Bahir Dar University, Bahir Dar, Ethiopia

**Keywords:** pooled, total fertility rate, sub-Saharan Africa, demographic, health survey

## Abstract

**Background:**

Even though the total fertility rate (TFR) has decreased significantly over the past decades in many countries, it has remained stable in sub-Saharan African (SSA) countries. However, there is variation among the sub-regions and inhabitants of SSA. Therefore, this study aimed to conduct a meta-analysis of demographic and health surveys (DHS) to estimate the pooled level of TFR in SSA and to depict sub-regional and inhabitant differences.

**Methods:**

The data source for this study was the standard Demographic and Health Survey datasets of 33 sub-Saharan African countries, accessed through www.meaasuredhs.com between 2010 and 2018. The point estimate of the total fertility rate with its corresponding standard error in each sub-Saharan African country was estimated using the *DHS.rates* R package. Using the point estimate of the TFR with the standard error of each country, the pooled estimate of the TFR was generated by the *metan* STATA command.

**Results:**

The study comprised 1,324,466 live births in total. The pooled estimate of sub-Saharan Africa's overall fertility rate was five children per woman (95% CI: 4.63–5.37). Consequently, the pooled estimate of total fertility for people living in urban and rural areas was 3.90 (95% CI: 3.60–4.21) and 5.82 (95% CI: 5.43–6.21) children per woman, respectively. In sub-group analysis, the pooled estimates of the TFR for the East African, Central African, Southern African, and West African regions, respectively, were 4.74, 5.59, 3.18, and 5.38 children per woman. Total fertility rates were greater in low-income nations (5.45), lower-middle-income countries (4.70), and high-middle-income countries (3.80).

**Conclusions:**

SSA has a relatively high total fertility rate. The regions of West and Central Africa have the highest overall fertility rate. The fertility rate is higher in countries with a large rural population and low income. Strategies should be developed to address this public health concern, especially in rural Central and Western Africa.

## Background

Many women want to have biological offspring (1, 2). The fertility rate is the total number of children a woman has during her reproductive period (1, 3). Fertility contributes to population growth in either a positive or negative way, depending on whether it is above and below the replacement level, respectively (3). According to early conventional demographic theory, high fertility results from a highly desired family size. People want their children to assist with the family. In addition, high child mortality can lead parents to have additional children to protect against loss or to replace lost children (4). The number of children desired by couples changes over time (5). In the last six decades since 1950, the total fertility rate (TFR) (the number of children each woman bears on average) has decreased worldwide, particularly in developed countries (6–8). The global fertility rate declined from 3.2 live births per woman in 1990 to 2.5 births in 2019. However, sub-Saharan Africa is the region with the highest fertility level (6), with no fertility reduction or only an incipient decline (9). In the industrialized world, fertility reached 2.8 births per woman in the late 1950s. It fell below the replacement level (1.7 per woman) in the late 1990s in Europe, North America, and Australia. Consequently, Japan reached below the replacement level in the late 1950s and has declined further (4, 10). However, in low-income countries, women have many babies (11–14). In low-resource countries, the demographic pattern is characterized by the coexistence of high infant and child mortality (15). Subsequently, in any region of the world, fertility in sub-Saharan African countries remained at the highest level (16). Studies indicated that, whenever fertility is high, maternal, infant, and child mortality are high (17). High fertility poses health risks for mothers and children, causes significantly slower economic growth, and exacerbates environmental degradation (18–21). As the fertility rate remains high, the youth dependency ratio also increases exponentially (22). Meanwhile, the proportion of reproductive-age women who were married or living in a union and used modern contraceptive methods was not >22% in SSA, compared with East Asia at 86% and Latin America and the Caribbean at 72% (22). Worldwide, in 2019, 50% of all women of reproductive age were using some form of contraception. However, in the same year, only 29% of women in sub-Saharan Africa were using some form of contraception (8). Due to the minimal utilization of contraceptives in Africa, particularly in SSA, women are exposed to unintended pregnancy. According to a recent report from the WHO, around 40% of pregnancies are unplanned (22, 23). Woman empowerment (increasing women's decision-making capacity) is identified as a key solution that can change the prevailing fertility and contraceptive utilization patterns in SSA (24–26). The level of fertility in SSA is projected to fall from 3.1 births per woman in 2010 to 2.1 births in 2050. Continued rapid population growth presents a challenge to achieving sustainable development, particularly in SSA (8). Although the DHS is a widely used source of estimates of fertility and mortality in low-income countries, estimates of the TFR and mortality can vary between different data sources. In a comparison study that compared DHS estimates of fertility with other estimates, the fertility rate computed from PMA2020 data showed a range of 5–22% difference compared to the result from DHS surveys (27).

Expanding access to contraceptives and reducing unmet needs for family planning can help decrease the TFR among reproductive-age group women in SSA (28). However, scarce data and inconsistent data sources create difficulties in monitoring progress in different regions of Africa for policy development and program planning. Although several data sources, including the DHS, have provided estimates of the TFR in SSA, no study has yet investigated the pooled fertility rate within the WHO sub-region (Eastern Africa, Southern Africa, Central Africa, and Western Africa) to allow for sub-regional comparison. Therefore, showing the pooled rate of TFR in sub-Saharan African countries among EDHS reports is critical for monitoring and evaluation.

## Methods

### Data sources

The DHS Program has been working with developing countries around the world to collect data about significant health issues, including fertility. The data are nationally representative, of high quality, follow standardized data collection procedures across countries, and have consistent content over time. We obtained raw data from www.meaasuredhs.com on all completed population-based surveys conducted under the DHS projects (29). The survey targeted women aged 15–49 years and men aged 15–59 years in randomly selected households in each country using a multi-stage sampling method. Detailed information was collected on the background characteristics of the respondents, including maternal health and child health, as well as harmful traditional practices (30). The source population included all mothers aged 15–49 years in the 3 years preceding the survey, excluding the month of the interview (1–36 months before the survey), in 33 SSA countries. The study population consisted of all reproductive-age mothers in the 3 years preceding the survey period in the selected enumeration areas (EAs) in each country. The data for this study were extracted from the birth record (BR) file in the standard DHS dataset for sub-Saharan African countries between 2010 and 2018. A total of 1,324,466 live births were included from sub-Saharan countries (11 East African, 6 Central African, 13 West African, and 3 South African countries) (Table 1).

**Table 1:** Number of live births from DHS in sub-Saharan Africa.

**Regions**	**Country**	**DHS year**	**Sample size**					
			**Total unweighted**	**Total weighted**	**Urban unweighted**	**Urban weighted**	**Rural unweighted**	**Rural weighted**
**SSA countries with recent DHS report from 2010 to 2018**								
East Africa countries	Burundi	2016/17	47,820	47,959	10,079	6,141	37,741	41,819
	Comoros	2012	14,769	14,778	6,270	4,904	8,499	9,873
	Ethiopia	2016	43,567	43,705	14,963	9,723	28,604	33,982
	Kenya	2014	87077	87611	33,169	36,603	50,908	51,007
	Malawi	2015/16	68524	68573	14,707	12,626	53,817	55,947
	Mozambique	2011	38030	38008	15,989	13,134	22,040	24,874
	Rwanda	2014/15	37,653	37,650	9,570	7,330	28,083	30,319
	Tanzania	2015/16	36,917	37,009	11,547	13,445	25,370	23,564
	Uganda	2016	51,266	51,338	12,260	13,907	39,006	37,431
	Zambia	2018	38,080	38,250	15,436	17,980	22,644	20,270
	Zimbabwe	2015	27,748	27,669	12,846	10,869	14,901	16,800
Central Africa countries	Angola	2015/16	40,013	39,931	24,829	27,743	15,184	12,188
	Cameroon	2011	42,934	42,970	21,678	23,194	21,256	19,776
	Chad	2014/15	49,143	49,150	11,816	11,608	37,327	37,542
	The DRC	2013/14	52,903	52,829	18,958	20,047	33,945	32,781
	R C	2011/12	30,350	30,323	9,918	20,788	20,432	9,535
	Gabon	2012	23,607	23,722	16,027	21,038	7,580	2,684
West Africa countries	Benin	2017/18	44,500	44,488	19,739	18,938	24,761	25,549
	Burkina Faso	2010	48,027	48,153	14,874	12,849	33,153	35,305
	Ivory Coast	2011/12	28,300	28,322	12,717	14,374	15,583	13,948
	Gambia	2013	28,544	28,602	12,611	16,077	15,933	12,525
	Ghana	2014	26,344	26,484	13,028	14,352	13,316	12,132
	Guinea	2018	29,736	29,672	10,985	11,123	18,752	18,550
	Liberia	2013	25,744	25,534	10,225	15,413	15,519	10,121
	Mali	2018	29,529	29,676	9,814	7,782	19,715	21,894
	Niger	2012	31,728	31,759	9,549	5,894	22,179	25,864
	Nigeria	2018	116,888	116,876	47,535	53,606	69,353	63,270
	Senegal	2010/11	43,861	44,058	17,256	21,814	26,604	22,244
	Sierra Leone	2013	45,643	45,850	18,315	16,192	27,328	29,658
	Togo	2013/14	26,831	26,877	10,227	12,265	16,604	14,612
Southern Africa countries	Lesotho	2014	18,346	18,463	6,132	6,844	12,214	11,619
	Namibia	2013	25,856	25,857	13,816	14,879	12,040	10,978
	South Africa	2016	24,188	24,284	13,745	16,446	10,443	7,838
Total sample size	1,324,466		1,326,430		500,630	529,928	820,834	796,499

### Eligibility

**Figure 1:**
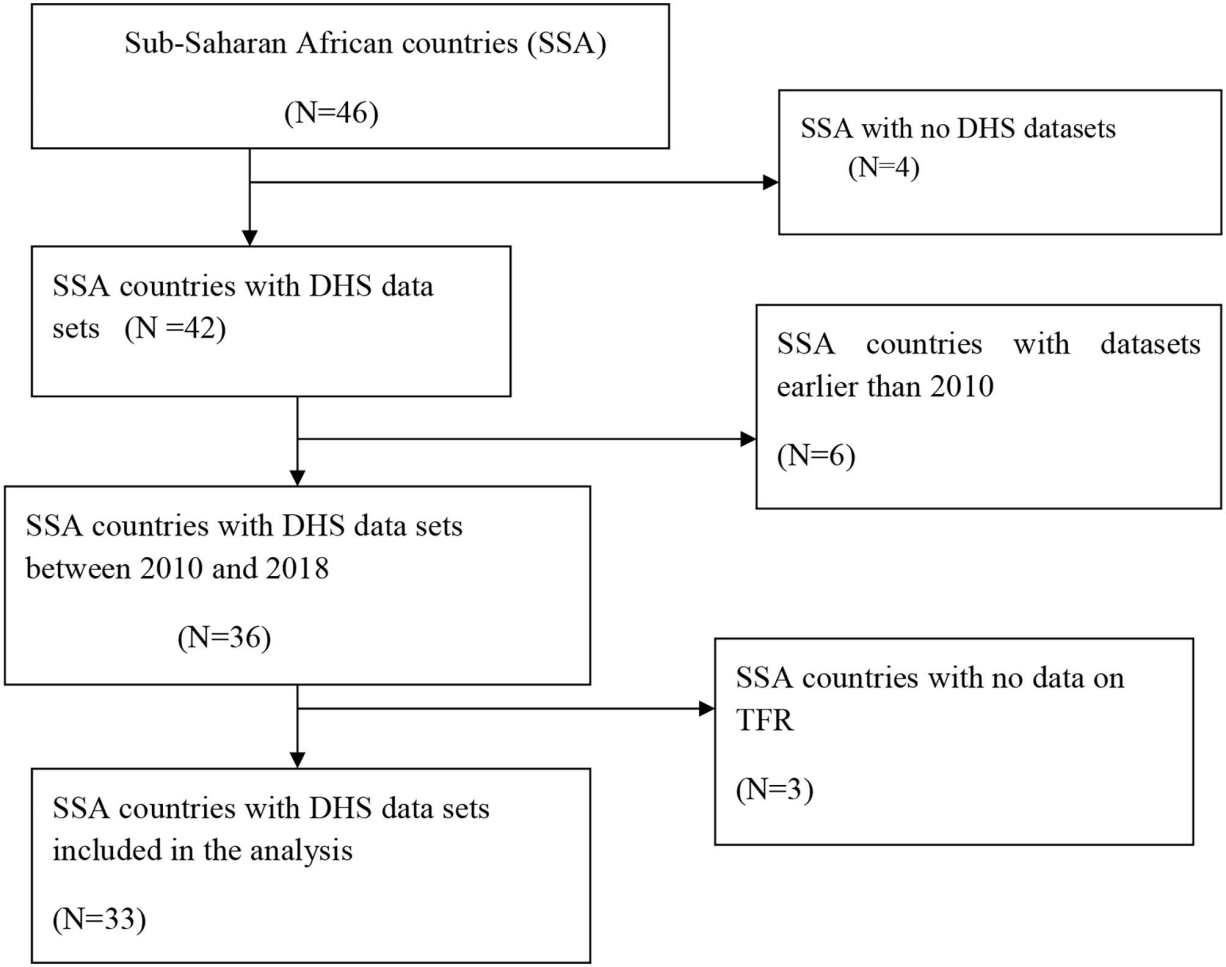
Flow chart for country selection.

Reproductive-age women aged 15–49 years in the 3 years preceding the survey in the selected enumeration areas of each country were included in this study. However, countries (the Central African Republic, Eswatini, Sao Tome and Principe, Madagascar, and Sudan) that did not have a DHS survey report after the 2010/2011 survey year were excluded. Three sub-Saharan countries (Botswana, Mauritania, and Eritrea) were excluded because the dataset was not publicly available (Figure 1).

The outcome variable of this study was the total fertility rate. The TFR is a hypothetical measure of women's fertility. It could be defined as the number of children born per woman if she experienced current age-specific fertility rates throughout her childbearing years and did not die, according to a current schedule of age-specific fertility rates, and not be subject to mortality. In standard DHS surveys, *ASFRa* is calculated for a reference period of 3 years preceding the survey for seven five-year age groups. Thus, the TFR can be written as follows:


$$\begin{array}{l} TFR=\sum_{a\varepsilon A}ASFRa/1,\;100,\\ \;A=(15-19,\;20-24,\;25-29,\;30-34,\\ \;35-39,\;40-44,\;45-49). \end{array}$$

### Data management and statistical analysis

The data were extracted using Microsoft excel and R Software. The point estimate of TFR with the standard error for each country was extracted from the individual record file (IR file) using the *DHS.rates* R package. In each country, along with fertility, the “fert” function estimates standard error (SE), relative standard error (RSE), and confidence interval (CI) for each rate (31). The methods of calculating the standard errors in the DHS rates package were in line with the DHS approach detailed in the DHS Sampling and Household Listing Manual. After extracting the point estimate and standard error, the pooled estimate of TFR (Table 2) was pooled using the “*metan”* STATA command. The pooled estimation of the TFR was determined with the random-effects model using DerSimonian-Laird weight. A subgroup analysis was conducted based on the sub-regions of sub-Saharan Africa, residence, and country income status.

**Table 2:** The TFR with their standard error in sub-Saharan African countries.

**Country**	**DHS year**	**TFR**	**standard error**	**Urban TFR**	**standard error**	**Rural TFR**	**standard error**
Burundi	2016/17	5.519	0.076	4.102	0.177	5.727	0.076
Comoros	2012	4.324	0.151	3.468	0.21	4.763	0.191
Ethiopia	2016	4.562	0.155	2.285	0.134	5.197	0.167
Kenya	2014	3.905	0.066	3.074	0.085	4.545	0.0078
Malawi	2015/16	4.433	0.075	3.025	0.146	4.746	0.072
Mozambique	2011	5.921	0.099	4.528	0.144	6.627	0.115
Rwanda	2014/15	4.165	0.067	3.565	0.16	4.308	0.072
Tanzania	2015/16	5.198	0.121	3.802	0.192	5.995	0.126
Uganda	2016	5.38	0.086	3.994	0.147	5.91	0.089
Zambia	2018	4.685	0.104	3.41	0.091	5.832	0.112
Zimbabwe	2015	4.024	0.091	2.994	0.111	4.701	0.102
Angola	2015/16	6.216	0.14	5.343	0.145	8.237	0.159
Cameroon	2011	5.088	0.103	3.977	0.109	6.395	0.121
Chad	2014/15	6.447	0.094	5.394	0.165	6.775	0.099
Democratic Republic of Congo	2013/14	6.566	0.117	5.423	0.179	7.27	0.127
Republic of the Congo	2011/12	5.11	0.109	4.481	0.13	6.461	0.133
Gabon	2012	4.103	0.125	3.861	0.122	6.125	0.341
Benin	2017/18	5.685	0.083	5.176	0.138	6.062	0.098
Burkina Faso	2010	5.991	0.099	3.919	0.146	6.738	0.078
Ivory Coast	2011/12	4.958	0.146	3.709	0.146	6.265	0.149
Gambia	2013	5.603	0.133	4.651	0.171	6.805	0.139
Ghana	2014	4.194	0.119	3.44	0.13	5.089	0.173
Guinea	2018	4.824	0.102	3.842	0.138	5.451	0.117
Liberia	2013	4.729	0.14	3.844	0.153	6.114	0.13
Mali	2018	6.281	0.126	4.874	0.188	6.775	0.142
Niger	2012	7.636	0.104	5.593	0.147	8.113	0.109
Nigeria	2018	5.288	0.067	4.498	0.089	5.944	0.082
Senegal	2010/11	4.984	0.118	3.911	0.142	6.039	0.126
Sierra Leone	2013	4.911	0.12	3.454	0.172	5.697	0.105
Togo	2013/14	4.781	0.111	3.666	0.114	5.722	0.136
Lesotho	2014	3.263	0.102	2.255	0.12	3.855	0.118
Namibia	2013	3.647	0.094	2.932	0.105	4.678	0.124
South Africa	2016	2.643	0.067	2.432	0.084	3.098	0.101

## Results

### The pooled TFR estimate in sub-Saharan Africa

**Figure 2:**
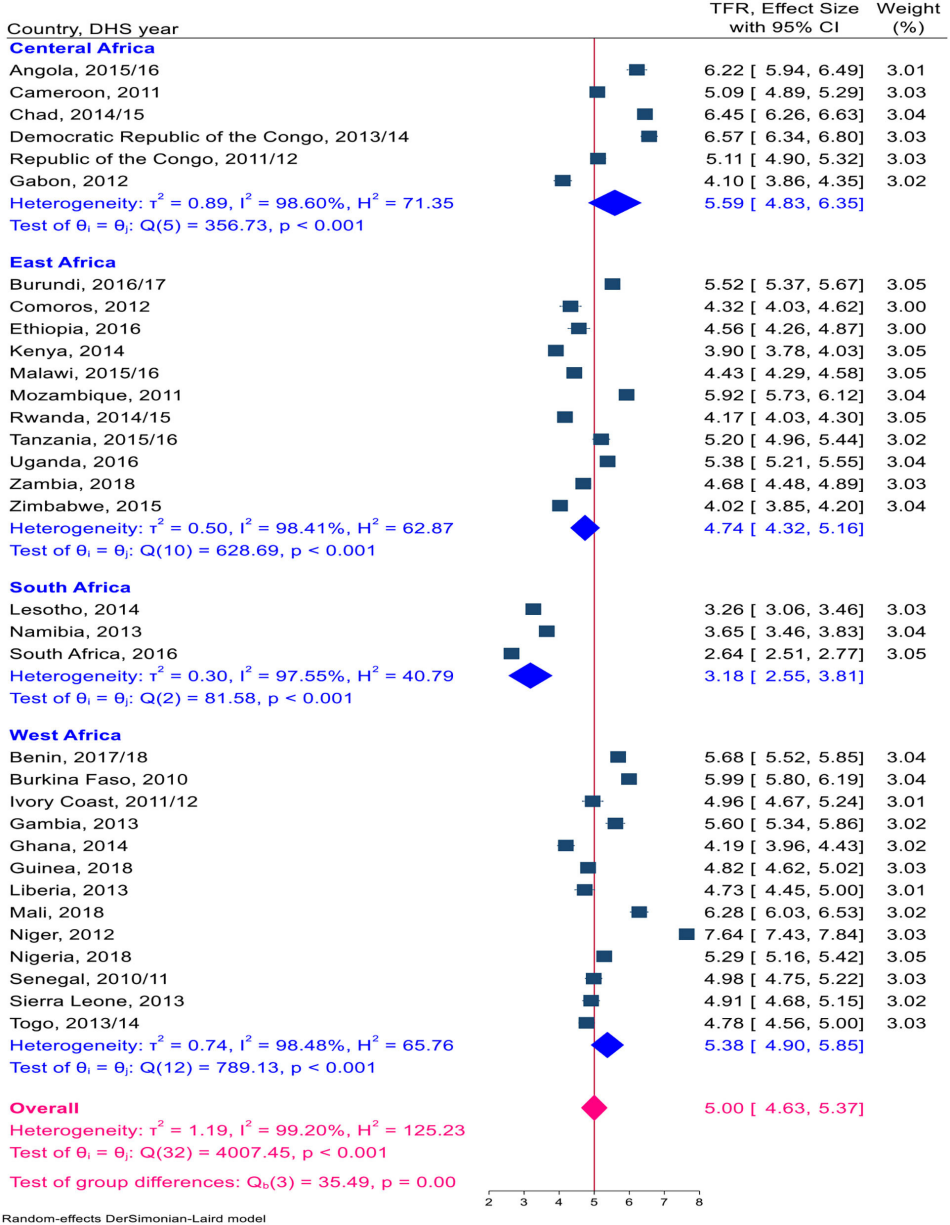
A Forest plot of the pooled estimate of TFR in SSA countries using the recent DHS 2010–2018, 2020.

**Figure 3:**
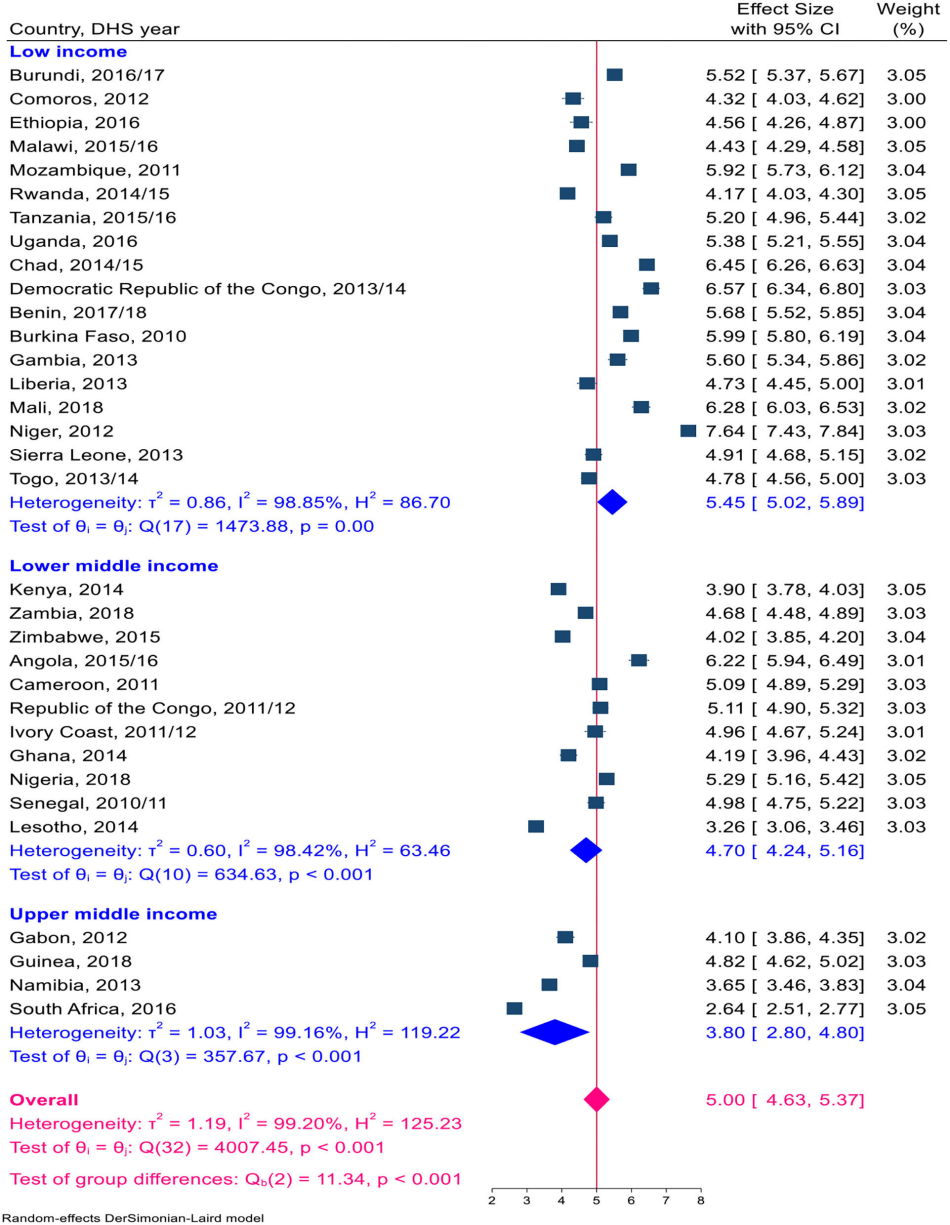
A Forest plot of the pooled estimate of TFR by country income across sub-Sharan African countries using the recent DHS between 2010–2018, 2020.

**Figure 4:**
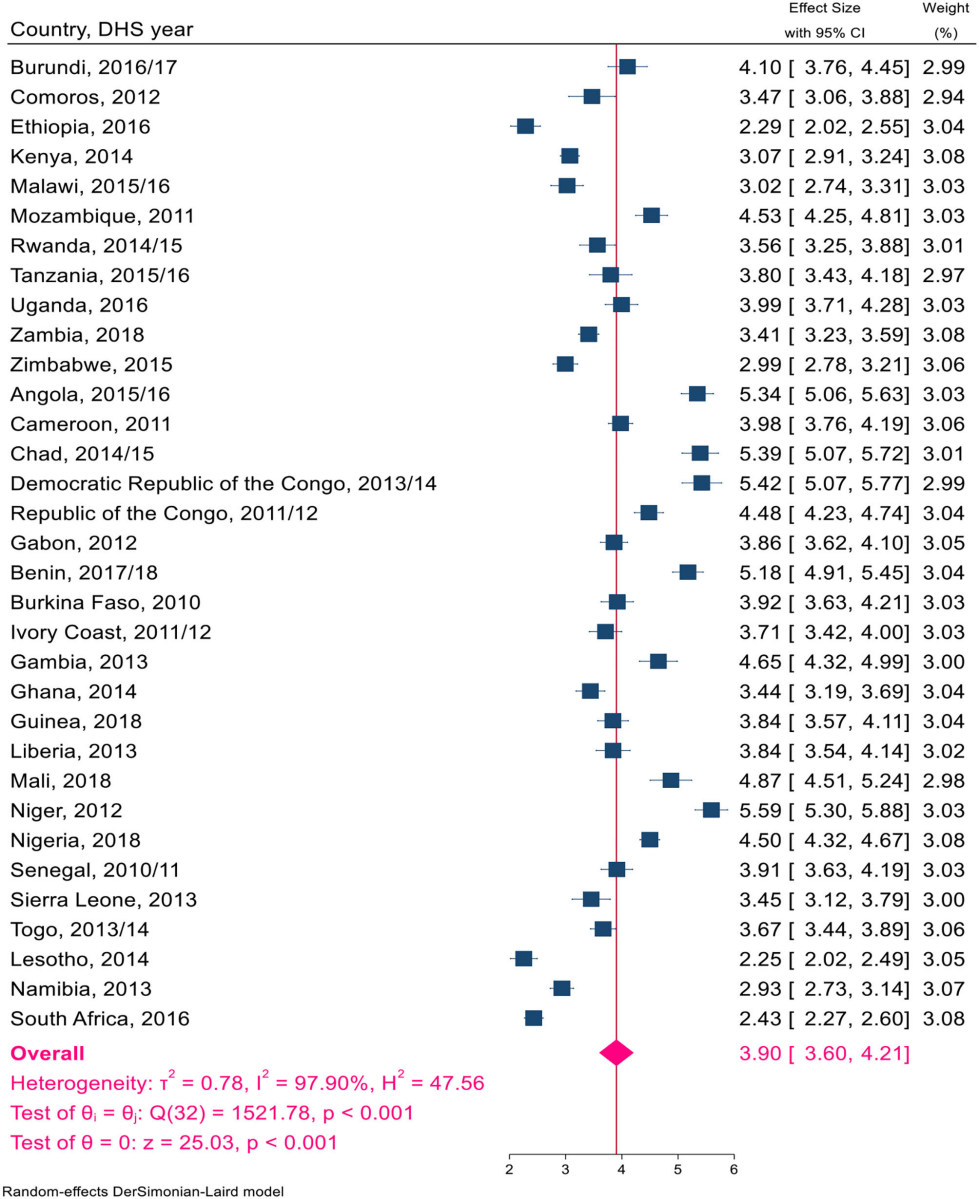
A Forest plot of the pooled estimate of TFR by urban residents in sub-Sharan African countries using the recent DHS between 2010–2018, 2020.

**Figure 5:**
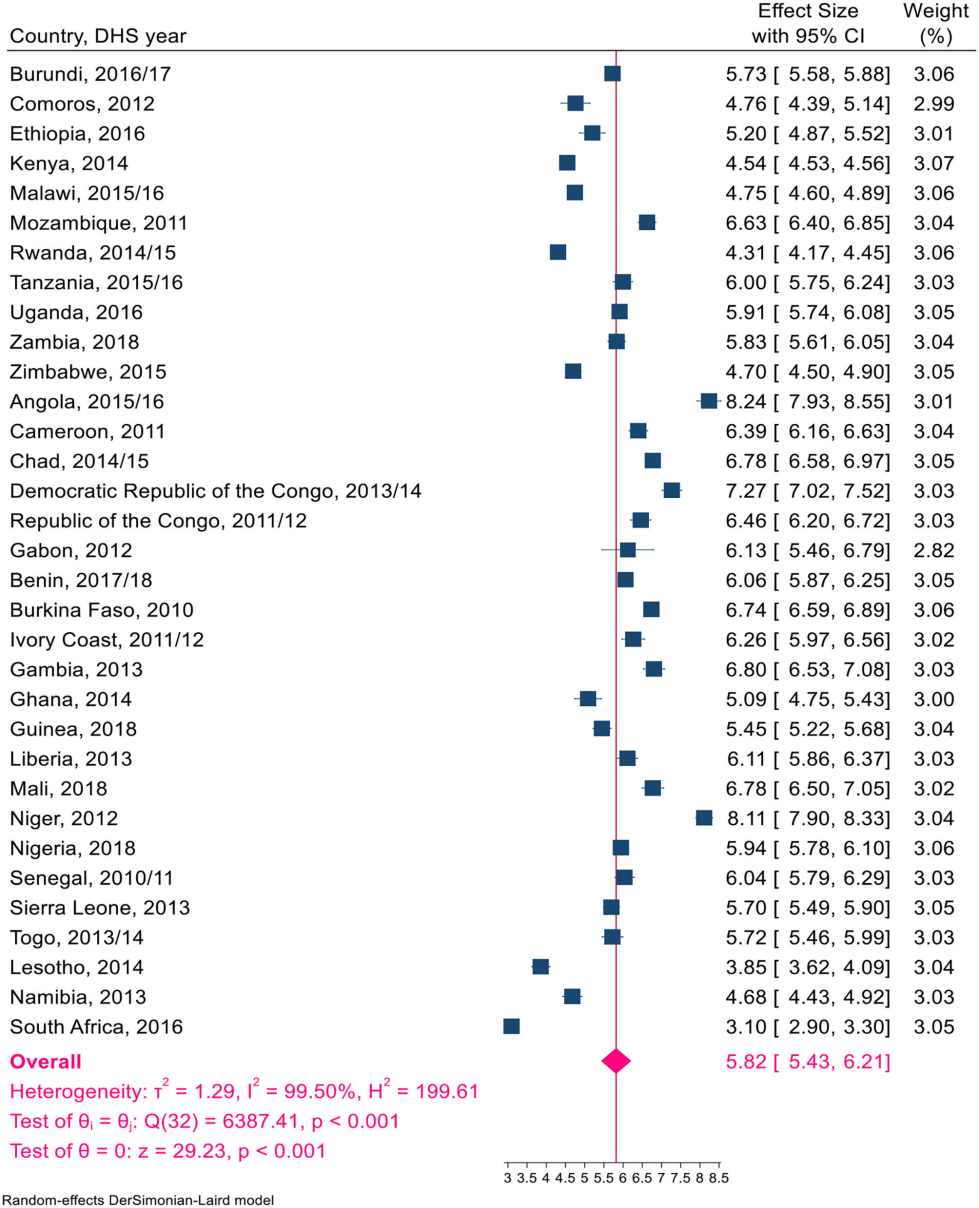
A Forest plot of the pooled estimate of TFR by rural inhabitants in SSA countries using the recent DHS between 2010–2018, 2020.

Overall, a total of 1,324,466 live births, with a minimum of 14,769 in Comoros and a maximum of 116,888 in Nigeria, were included in this study (Table 1). The pooled TFR estimate for sub-Saharan African countries was calculated. The pooled TFR estimate for 33 countries in sub-Saharan Africa was five children per woman (95% CI: 4.63–5.37). The pooled TFR estimates for East African countries were 4.74 (95% CI: 4.32–5.16) children per woman, 5.59 (95% CI: 4.83–6.35) children per woman in Central Africa, 3.18 (95% CI: 2.55–3.81) children per woman in Southern Africa, and 5.38 (95% CI: 4.49–5.85) children per woman in West Africa (Figure 2). The pooled TFR estimates for low-, middle-, and upper-middle-income countries were 5.45 (95% CI: 5.02–5.89), 4.70 (95% CI: 4.24–5.16), and 3.80 (95% CI: 2.80–4.80), respectively (Figure 3). Consequently, the pooled TFR estimate for urban and rural inhabitants was 3.90 (95% CI: 3.60–4.21) and 5.82 (95% CI: 5.43–6.21), respectively (Figures 4, 5).

### Discussions

The fertility decline in SSA countries has been relatively steady and occurred later compared to other regions in the world. Its age-specific fertility rate also showed substantial variation among countries (3, 32, 33). The pace of decline varies considerably across countries; as a result, the median pace change in SSA countries (0.03 per year) is less than one-third the pace in other regions (0.13 per year) (34). Throughout resource-constrained countries, a considerable proportion of women who did not want to become pregnant were not using contraception. There could be multiple barriers to using contraception that contribute to this “unmet need” for contraception. Similarly, there are noticeable differences among some regions in Africa. The demand for and “met need” for family planning is higher in eastern and southern African countries than in central and western African countries, which is a major factor in the increase in fertility.

In this study, the overall pooled estimate of the TFR from DHS data in SSA was five per woman. Even though the TFR has fallen significantly in the past decades in many countries, it has remained stable (or “stable population”) at around 6,400 children per 1,000 women in 1990 (3) in SSA countries. According to a report from the World Health Organization 1 year ago (2019), the TFR in SSA was 4,600 per 1,000 women (8, 32). In the same time period, the average number of children a woman would have by the end of her childbearing years also declined in Latin America and the Caribbean (from 3.3 to 2.4), in Central and Southern Asia (from 4.3 to 2.4), in Eastern Asia (from 2.5 to 1.8), and in Northern Africa (from 4.4 to 2.9). There is a discrepancy between the TFR of SSA countries reported by the WHO in 2019 and the current (2020) pooled DHS reports.

A possible justification for this discrepancy might be the data source that the two studies used. As mentioned previously, there is a considerable decrease in the TFR from time to time in different regions of the world. However, the decrement is steady in SSA. There are several reasons why SSA lacks a marked decline in total fertility, including political instability, poverty, a lack of government commitment to female education, a weak healthcare system, and cultural beliefs that view children as sources of income; in almost all African countries, early marriage is taboo (35, 36). In contrast, when women delay childbearing and the mean age of childbirth increases, the TFR decreases because of the reduced number of births over a woman's reproductive years (10). Compared to our study, in India, the TFR was 2.2 per woman among sampled registration systems in 22 states in 2017, which was close to the replacement level; this decrease was attributed to factors such as higher education, increased mobility, late marriage, and financial independence for women (37).

In this study, the distributions of total fertility in the sub-region of SSA countries are not similar. Consequently, the pooled estimates of TFR in Eastern Africa, Southern Africa, Central Africa, and Western African countries were 4.74, 3.18, 5.59, and 5.38, respectively. Countries within each sub-region have experienced sociodemographic, socio-political, economic, and cultural events that could influence total fertility variations. In Central Africa and Western African countries, the TFR is higher than the pooled level. This rise could be because >50% of women in these regions experience their first marriage, sexual initiation, and the birth of their first child by the age of 20 (33). In contrast, in China, only 2% of children are born to teenage mothers (38, 39).

Besides, West and Central African countries have low rates of modern contraceptive use, with some of the lowest contraceptive prevalence rates in the world in two sub-regions of Africa (40); this could be because family planning is not accepted by certain religious and social doctrines and because these regions tend to have a preference for large families (33). Countries such as almost all of central Africa except Gabon, Mozambique in East Africa, and Niger in West African countries reported the highest TFR within each sub-region. Meanwhile, in this study, among 33 SSA countries, Niger reported the highest total fertility rate. However, South Africa's TFR is significantly lower than in other sub-regions of sub-Saharan Africa. In the meantime, countries in southern Africa reported the highest level of contraceptive use, followed by countries in East Africa. Modernization, urbanization, school-based education, and male involvement were higher in Southern Africa than in other sub-regions of SSA countries (40).

Ascertaining fertility levels by residence is crucial for a better understanding of contemporary demographic changes in developing countries. The pooled fertility level was significantly higher in rural inhabitants than in urban inhabitants, at 3.90 and 5.82 per woman, respectively. These findings were also consistent with some studies (41, 42). Because there are known variations in social, economic, demographic, and health characteristics across urban and rural settlements (43–45). Another sub-group in which we saw the variation in total fertility was income. This study showed a significant variation in total fertility in upper-middle-income countries and low-income countries. Because in upper-middle-income countries, the age at which women first give birth is delayed, which influences the number of children a woman will conceive in her lifetime, and the proportion of women progressing from parity to the next parity was high (15-9). In high-income agricultural societies, parents tend to need relatively fewer children (using modern contraceptives). In contrast, in lower-income societies, women want many children, and adolescents are more likely to experience unwanted and poorly timed pregnancies (4, 46, 47).

## Conclusions

This pooled estimate revealed that the TFR in sub-Saharan countries is high, with the highest TFR being reported in central and west African countries. The fertility rate of rural dwellers was higher compared to those living in urban areas. Decreasing total fertility can indirectly lead to a decrease in maternal and child mortality. However, the decrease was steady in SSA countries. Therefore, appropriate efforts should be made to reduce the TFR in sub-Saharan countries, particularly in the Central and Western African sub-regions and among rural inhabitants. The government and health professionals should work toward meeting the reproductive and maternal health needs of the population.

## Data availability statement

The datasets presented in this study can be found in online repositories. The names of the repository/repositories and accession number(s) can be found in the article/supplementary material.

## Author contributions

DT and ST were involved in this study from its inception through the design, acquisition, analysis, interpretation of data, and manuscript drafting. All authors contributed to the conception and design, analysis, and interpretation of the data and reviewed and revised the manuscript for important intellectual content, and also gave final approval for the version to be published and are accountable for all aspects of the study.
